# Prevalence of disability in a composite ≥75 year-old population in Spain: A screening survey based on the International Classification of Functioning

**DOI:** 10.1186/1471-2458-11-176

**Published:** 2011-03-23

**Authors:** Javier Virués-Ortega, Jesús de Pedro-Cuesta, Manuel Seijo-Martínez, Pedro Saz, Fernando Sánchez-Sánchez, Fermina Rojo-Pérez, Fernanda Rodríguez, Raimundo Mateos, Pablo Martínez-Martín, Ignacio Mahillo, Jordi Gascon-Bayarri, Josep Garre-Olmo, Francisco Jose García, Gloria Fernández-Mayoralas, Felix Bermejo-Pareja, Alberto Bergareche, Javier Almazan-Isla, Jose Luis del Barrio

**Affiliations:** 1 Research Network in Neurodegenerative Diseases (CIBERNED), Carlos III Institute of Health, Madrid, Spain; 2 Department of Applied Epidemiology, National Centre for Epidemiology, Carlos III Institute of Health, Madrid, Spain; 3 Neurology Service, Hospital do Salnés, Pontevedra, Spain; 4 Department of Medicine and Psychiatry, Zaragoza University and CIBERSAM, Zaragoza, Spain; 5 Neurology Department, 12 de Octubre University Teaching Hospital, Madrid, Spain; 6 Center for Human and Social Sciences, Spanish Council for Scientific Research (CSIC), Madrid, Spain; 7 Neurology Unit, Segovia General Hospital, Segovia, Spain; 8 Psychiatry Department, University of Santiago de Compostela and Psychogeriatrics Unit, Santiago de Compostela University Hospital, Santiago de Compostela, Spain; 9 Dementia Diagnosis & Treatment Unit, Neurology Department, Bellvitge University Teaching Hospital, Barcelona, Spain; 10 Dementia Unit, Santa Caterina Hospital, Gerona, Spain; 11 Virgen del Valle Geriatric Hospital, Toledo, Spain; 12 Neurology Department, Donostia Hospital, Bidasoa-Hondarribia Hospital, Guipúzcoa, Spain

## Abstract

**Background:**

The prevalence and predictors of functional status and disability of elderly people have been studied in several European countries including Spain. However, there has been no population-based study incorporating the International Classification of Functioning, Disability and Health (ICF) framework as the basis for assessing disability. The present study reports prevalence rates for mild, moderate, and severe/extreme disability by the domains of activities and participation of the ICF.

**Methods:**

Nine populations surveyed in previous prevalence studies contributed probabilistic and geographically defined samples in June 2005. The study sample was composed of 503 subjects aged ≥75 years. We implemented a two-phase screening design using the MMSE and the World Health Organization-Disability Assessment Schedule 2^nd ^edition (WHO-DAS II, 12 items) as cognitive and disability screening tools, respectively. Participants scoring within the positive range of the disability screening were administered the full WHO-DAS II (36 items; score range: 0-100) assessing the following areas: Understanding and communication, Getting along with people, Life activities, Getting around, Participation in society, and Self-care. Each disability area assessed by WHO-DAS II (36 items) was reported according to the ICF severity ranges (No problem, 0-4; Mild disability, 5-24; Moderate disability, 25-49; Severe/Extreme disability, 50-100).

**Results:**

The age-adjusted disability prevalence figures were: 39.17 ± 2.18%, 15.31 ± 1.61%, and 10.14 ± 1.35% for mild, moderate, and severe/extreme disability, respectively. Severe and extreme disability prevalence in mobility and life activities was three times higher than the average, and highest among women. Sex variations were minimal, although life activities for women of 85 years and over had more severe/extreme disability as compared to men (OR = 5.15 95% CI 3.19-8.32).

**Conclusions:**

Disability is highly prevalent among the Spanish elderly. Sex- and age-specific variations of disability are associated with particular disability domains.

## Background

The increasing survival rate from chronic diseases and decreasing birth rate are making Spain one of the fastest ageing societies in the world [1,2]. The proportion of the population over 65 years of age doubled during the last 30 years and is expected to double again by 2050 [3]. The 1999 National Survey on Disability, Impairments and Health indicated that 59% of individuals with disabilities in Spain were aged over 65 years. Moreover, 33% of those over 65 were disabled as identified in this survey [4]. Disability among the elderly people represents a major public health concern in Spain. National prevalence rates of disability and a quantitative approach to disability determinants are needed for service development and evidence-based health decision-making with regard to this population.

Over the last few decades, researchers have used a variety of approaches to disability measurement. These varied strategies have hampered comparability across studies and have rendered highly variable epidemiological conclusions on the distribution of disability [5]. Previous enquiries have frequently focused on dependence for activities of daily living (ADL, e.g., eating, moving around the home; cf., Katz index, Barthel index), and sensory and cognitive functions [4,6]. By contrast, the International Classification of Functioning, Disability and Health (ICF) [7] incorporates a multifactorial approach to disability with two core components: limitations in activities and participation, and changes in body structure and functions. Under the ICF model diseases, environmental factors, and personal characteristics can all function as determinants of disability. According to this framework, an individual's functional status results from the interaction between the health problems and the physical, social, and psychological context of the individual.

The ICF provides the basis to develop disease-specific disability profiles [8]. Moreover, the ICF facilitates the identification of targets in rehabilitation, assessment of intervention outcomes, and social and health service planning [9]. For instance, ICF-based models of assessment, as the World Health Organization Disability Assessment Schedule 2^nd ^Edition for epidemiological use (WHO DAS-II) [10], pinpoint specific spheres of the individuals personal and social functioning, which may be linked to specific needs of social support. Along these lines the ICF checklist for clinical use [11] provides a multi-faceted classification of the components of disability, the impact of environmental aids and the composition of the package of services that may best suit the needs of a particular individual.

The ICF scheme opens up the possibility of cross-national and multi-dimensional assessment of disability, thereby offering a more extensive picture of which aspect may be affected for any given individual or cohort. The WHO-DAS II has been developed under the ICF scheme and may constitute an effective tool for epidemiological surveys. WHO-DAS II is well suited for prevalence studies implementing door-to-door designs given that both the screening (12 items) and the full (36 items) versions of this instrument are available [12,13]. Traditional approaches to disability assessment incorporate items relevant to disability, particularly for clinical use in nursing (e.g., Katz index, Barthel index). However, they frequently amalgam items on function (e.g., digestive and urinary function, sphincter control, motor function) without any indication of impact on social functioning. By contrast, WHO-DAS II provides a systematic approach to various social and functional domains of disability, which are assessed separately over a single 5-point Likert scale. As an additional advantage, WHO-DAS II has been designed specifically for epidemiological use being available in various languages and formats [14].

There have been no previous population-based studies on health and functioning in the Spanish elderly population under the ICF framework. The ICF system may provide a more comprehensive framework to disability witch could potentially become a standard for psychological assessment [15].

The goal of the present study was to obtain prevalence estimates of disability levels in a Spanish elderly population using WHO-DAS II (12 items) as a screening tool, WHO-DAS II (36 items) as the basis for a multi-faceted assessment of disability, and ICF disability severity ranges to define cases of mild, moderate, and severe/extreme disability.

## Methods

### Study population

**Figure 1:**
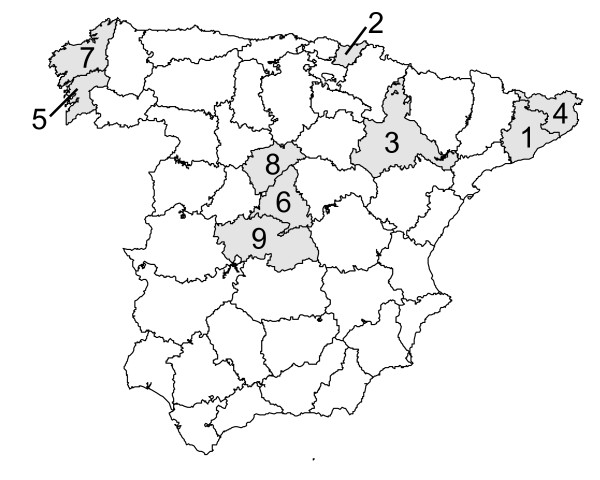
**Locations participating in the study**. 1. El Prat de Llobregat (Barcelona), n = 59; 2. Irún-Hondarribia (Guipúzcoa), n = 57; 3. Zaragoza, n = 31; 4. Gerona, n = 75; 5. Isla de Arosa (Pontevedra), n = 53; 6. Getafe (Madrid), n = 98; 7. Santiago de Compostela (La Coruña - Corunna), n = 33; 8. Cantalejo (Segovia), n = 24; 9. Toledo, n = 73.

Participants were part of a recent Spanish epidemiological multi-population study composed of nine probabilistic and geographically defined samples conducted in June 2005 in Spain (Figure 1). Co-investigators in charge of recruitment in each location were asked to contribute an average of 60 participants aged 75 years of more. Details on the characteristics of the resulting sample including its geographical distribution are available in Virues-Ortega et al. [16].

### Sampling procedure

We conducted a power analysis for cross-sectional designs [17]. The expected prevalence used during power analysis was that of individuals 70 years of age or more reported by de Pedro-Cuesta [18] (i.e., 707/12,232). Precision was set to 2%. Power analysis indicated that a sample size of 523 or more participants was required.

We requested from the principal investigators of the original surveys a census-based random sample of individuals aged 75 years or more from the population originally surveyed in their respective prevalence studies. Sampling ended when an average of 60 participants per group was reached. We used nationwide age- and sex-specific mortality rates for the birth cohorts under study in order to estimate the number of participants to be sampled for each location [19]. Mortality was proportional to the delay from the original survey and so were the number of participants to be sampled. Groups used their original census-based sampling procedure to avoid selection bias. In locations with very limited number of survivors a new geographically-defined sample was obtain from selected city neighbourhoods. This approach ensured that the main attributes of all sub-samples in terms of environment, living arrangements and residential status were represented. Cohorts included individuals living in their homes and also those in residential care and were drawn from rural and urban areas. Losses resulted from dead and inability to locate individuals surveyed in earlier studies, circumstances unlikely to induce specific selection biases in a geographically-defined prevalent sample. Death excludes individuals from the pool of survivors. Non-located individuals (due to death or change of residence outside the geographic residence) shall also be considered outside the sampling frame.

### Study design

The screening phase comprised the administration of WHO-DAS II (12 items) to all eligible participants. This instrument was used as the basis for screening participants before further disability assessments were conducted. This instrument has been recently validated with the Spanish population showing optimal internal consistency (Cronbach alpha = 0.89) [20]. Direct scores range from 0 to 48, with higher scores indicating greater disability. Given that the predictive value of specific WHO-DAS II (12 items) cut-off points has not been established for this population, all participants scoring above 0 passed on to the assessment phase in which the full 36-item version of WHO-DAS II was administered.

In view of the fact that dementia is the primary health condition contributing to disability in the elderly [20] and that it is highly under-diagnosed in Spain [21], a simultaneous cognitive screening procedure was conducted. As indicated by Pressley et al. [22], a multi-source approach may maximize the detection of dementia and therefore be an optimal strategy for studying the prevalence and determinants of dementia and disability. The Spanish version of the Mini-Mental State Examination (MMSE) [23,24] was used for screening purposes (with a cut-off of 24). Further details on the dementia diagnosis procedure and study design are available elsewhere [16].

The study was approved by the Carlos III Health Institute Human Subjects Review Board (ref. no. CEI PI 4_2009), and written informed consent was given by all participants in accordance with the Helsinki Declaration. Participants were visited twice at their homes or nursing homes. During the first visit, cognitive screening through the Spanish version of the MMSE was administered [16]. In addition, health status was determined through direct medical examination and a structured medical questionnaire. Interviewers had access to participants' hospital and primary care medical records. A physician specialized or undergoing specialized training in neurology, psychiatry, or geriatrics conducted this first visit. On the second visit, a psychologist, physician or nurse evaluated the participant's functioning and disability. Interviewers underwent formal training in all assessment procedures used during the study.

### Disability assessment (WHO-DAS II)

WHO-DAS II (36-item) is a self-reported scale covering six disability domains assessed over the 30-day period preceding administration [25]: Understanding and communication (UAC); Getting around (GAR); Self-care (SCA); Getting along with people (GAP); Life activities (LAC); and Participation in society (PSO) (see definitions in Table 1). Items are answered in a 5-point Likert format (1: none; 5: extreme), which grades the difficulty experienced by the participant in performing a given activity. The preliminary scoring rules developed by the WHO were used as provided by the WHO Spanish Official Group (Dr. Vazquez-Barquero, Universidad de Cantabria, personal communication). We obtained summary-index and domain scores by summing across items and transposing the result to a 100-point scale. Items D5.5 thru D5.8 (work) were omitted in all subjects, as most individuals in our sample were not gainfully employed. Similarly, items D5.2-D5.5 (life activities) were not applicable in a sub-sample of 37 positively screened participants, as they had no household activities assigned. In addition, item D4.5 (sexuality) was also excluded, owing to an unusually high proportion of missing values (47.2%). WHO-DAS II proxy informant version was administered for participants who were unable to answer questions due to cognitive or motor disabilities. WHO-DAS II subscales and summary indices were coded using the ICF disability categories; namely: No problem (0%-4%); Mild problem (5%-24%); Moderate problem (25%-49%); Severe problem (50%-95%); and Extreme problem (95%-100%) (see definitions in Table 1).

**Table 1:** Qualitative definition of WHO-DAS II subscales and ICF disability levels

WHO-DAS II Subscales	Definition ^a^
UAC (6, 0.81) ^b^	Difficulty concentrating on something for more than 10 min and learning new tasks.
GAR (5, 0.88)	Difficulty standing for long periods, moving around the house and getting out of the house.
SCA (4, 0.71)	Difficulty in bathing, getting dressed, feeding and being independent while being alone.
GAP (5, 0.77)	Difficulty in social activities such as starting and maintaining a conversation, dealing with unknown people and maintaining or making new friends.
LAC (4. 0.96)	Difficulty in performing instrumental activities quickly and effectively, particularly household duties.
PSO (8, 0.95)	Difficulty joining community activities such as festivities or religious. Lack of self-confidence due to health problems.
Summary Index (32, 0.93)	Average score across the six domains.

ICF Disability levels	Definition ^c^

No problem (0-4%)	No problem as measured by standardised instruments (5% error allowed).
Mild disability (5-24%)	Problem that expands up to a fourth of the time or, alternatively, the first fourth of the score range of a standardised instrument on self-reported difficulty for an activity/participation.
Moderate disability (25-49%)	Problem that expands up to a half of the time or, alternatively, half of the score range of a standardised instrument on self-reported difficulty for an activity/participation.
Severe disability (50-95%)	Problem that expands up to 95% of the time or, alternatively, a score on a standardised instrument on self-reported difficulty for an activity/participation up to 95% of the score range.
Extreme disability (96-100%)	Complete problem as measured by standardised instruments; 5% error allowed.

UAC = Understanding and communication; GAP = Getting along with people; LAC = Life activities; GAR = Getting around; PSO = Participation in society; SCA = Self-care. Activity = execution of a task or action by an individual; Participation = involvement in a life situation; Problem: self-reported difficulty/number of days with disability over the last month in the actual performance or the abstract capacity (no environmental aids) complete an activity.

^a^According to Vázquez-Barquero et al. 2006, p. 88. ^b^Number of items, Cronbach's α. ^c^According to WHO, 2001, p. 134.

### Data analysis

Cases of mild, moderate, and severe/extreme disability were identified to obtain age- and sex-specific disability prevalence and standard errors, according to WHO-DAS II domains and summary index. Standard errors were calculated from the binomial distribution where the product of the proportion multiplied by N was below 5 [26]. In addition, we obtained age-standardized prevalence figures according to the European standard population weights (0.50, 0.25 and 0.25 for 75-79, 80-84 and ≥85 years of age, respectively [27]). Direct prevalence adjustments are used to compensate the disparity in the age structure across genders between the population and the sample under study. Finally, we used the sex and age distribution for those aged 75 years and over in the Spanish population to estimate the number of individuals with severe/extreme disability in the whole country (Spanish general population of ≥75 years of age for 2009: 3,846,132 inhabitants; source: http://www.ine.es). Binary logistic regression was used to obtain odds ratios (OR) and 95% confidence intervals (95% CI) in order to compare risk for disability status across genders, age groups, and disability domains.

## Results

The final sample comprised 546 participants of which 440 were positive to the disability screening and 106 scored 0 in WHO DAS II (12 items). Of this total, 503 had complete datasets (mean age: 82.0, SD: 4.8; 62.6% women). Most participants were of rural-mixed, urban, and sub-urban origin, mid or mid-low social class, and had a low level of education. Two-thirds of the sample was living in rural areas (municipality size ≤ 10000 inhabitants). The actual proportion in the whole country is slightly inferior (≈60%; source: Spanish National Institute of Statistics, http://www.ine.es/censo_accesible/es/seleccion_colectivo.jsp?fType=1), which may have bias slightly our results in terms of disability. The socio-demographic characteristics of the study population are shown in Table 2. Prevalent health conditions and morbidity of study sample is described in detail on Table S1 (online additional file 1).

**Table 2:** Characteristics of study participants (n = 503)

	% (n)
Age (years)	
75 - 79	
Women	24.85 (125)
Men	15.31 (77)
80 - 84	
Women	16.90 (85)
Men	13.12 (66)
≥ 85	
Women	20.87 (105)
Men	8.95 (45)
Self-reported social status	
Low	10.14 (51)
Middle-low	30.42 (153)
Middle	51.29 (258)
Middle-high	6.96 (35)
High	1.19 (6)
Education	
Illiterate	9.74 (49)
Primary incomplete	41.55 (209)
Primary complete	34.19 (172)
Some secondary or higher	14.51 (73)
Municipality size	
1 - 10,000 inhabitants	65.40 (329)
> 10,000 inhabitants	34.60 (174)
Cognitive status (MMSE score)	
< 24	19.48 (98)
≥ 24	80.52 (405)

**Figure 2:**
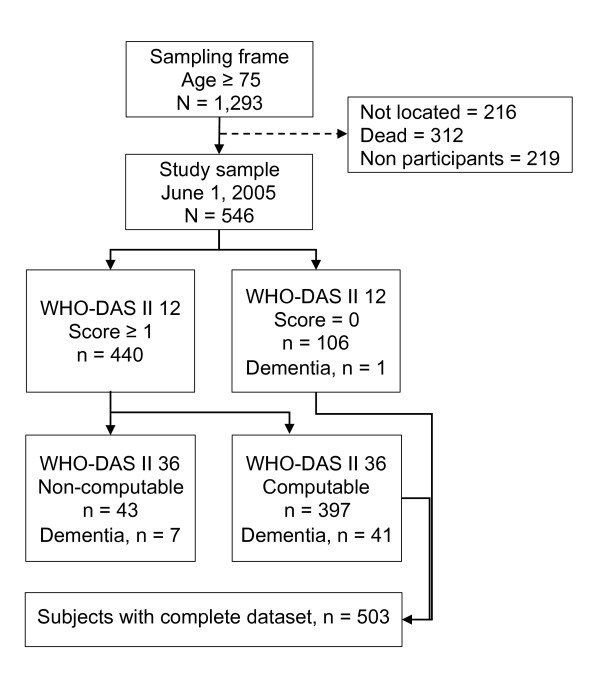
Sample attrition

Figure 2 shows a flow chart portraying the sample attrition. A total of 503 computable datasets were available. Non-computable scores were due to incomplete information and lack of collaboration during the second visit (n = 43). A total of 49 participants had dementia; among them only one score 0 on the disability screening. Seven out of 48 dementia cases scoring within the positive range of the disability screening did not have computable scores at the WHO-DAS II (36 items).

### Disability prevalence

**Figure 3:**
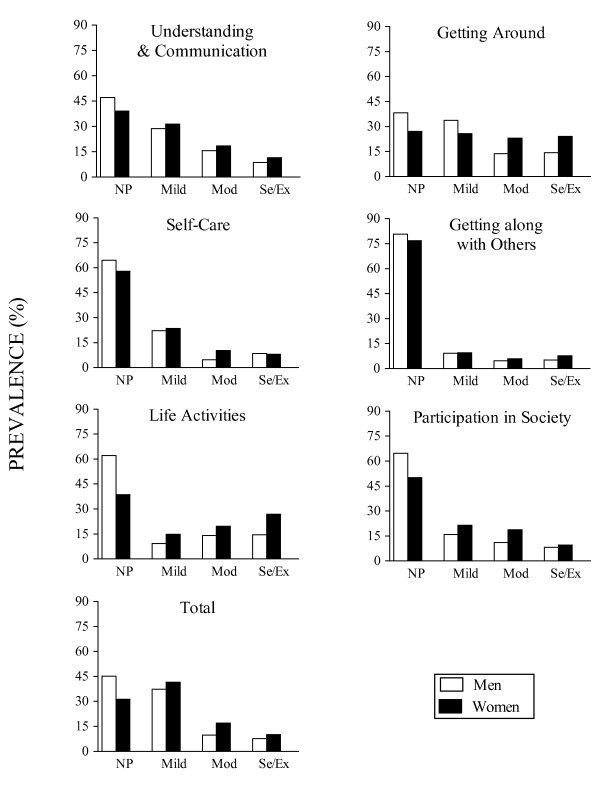
**Raw and age-standardised (European standard) prevalence of disability levels according to ICF disability ranges established through the scores of WHO-DAS II 36 items (n = 503) **. NP: No problem; Mild: Mild disability; Mod: Moderate disability; Se/Ex: Severe or extreme disability

Age-standardized prevalence figures are presented in Figure 3 (see age- and sex-specific prevalence of disability in Table 3). Table 4 present binary logistic models of the various disability domains across age intervals and gender taking individuals with severe/extreme disability as cases. Due to the limited number of individuals scoring within the range of extreme disability, severe and extreme disability categories were collapsed into a severe/extreme disability category. Total age-standardized prevalence rates for disability (age ≥75) based on the WHO-DAS II summary index was: 39.17 ± 2.18% (mild disability); 15.31 ± 1.61% (moderate disability); and 10.14 ± 1.35% (severe/extreme disability).

**Table 3:** Age- and sex-specific prevalence of disability levels (n = 503)

	UAC	GAR	SCA	GAP	LAC	PSO	Summary index	
	% ± SE	% ± SE	% ± SE	% ± SE	% ± SE	% ± SE	n	% ± SE
MILD								
Both sexes								
75-79	30.69 ± 3.25	31.19 ± 3.26	21.78 ± 2.90	8.42 ± 1.95	15.79 ± 2.65	20.30 ± 2.83	86	42.57 ± 3.48
80-84	28.48 ± 3.67	32.45 ± 3.81	19.21 ± 3.21	9.93 ± 2.43	11.43 ± 2.69	17.22 ± 3.07	62	41.06 ± 4.00
≥85	31.33 ± 3.79	19.33 ± 3.22	30.00 ± 3.74	10.67 ± 2.52	8.09 ± 2.34	19.33 ± 3.22	49	32.67 ± 3.83
Male								
75-79	27.27 ± 5.08	31.17 ± 5.28	22.08 ± 4.73	6.49 ± 2.81	8.96 ± 3.49	16.88 ± 4.27	29	37.66 ± 5.52
80-84	28.79 ± 5.57	39.39 ± 6.01	18.18 ± 4.75	10.61 ± 3.79	8.77 ± 3.75	12.12 ± 4.02	24	36.36 ± 5.92
≥85	31.11 ± 6.90	33.33 ± 7.03	26.67 ± 6.59	13.33 ± 5.07	10.53 ± 4.98	17.78 ± 5.70	17	37.78 ± 7.23
Female								
75-79	32.80 ± 4.20	31.20 ± 4.14	21.60 ± 3.68	9.60 ± 2.63	19.51 ± 3.57	22.40 ± 3.73	57	45.60 ± 4.45
80-84	28.24 ± 4.88	27.06 ± 4.82	20.00 ± 4.34	9.41 ± 3.17	13.25 ± 3.72	21.18 ± 4.43	38	44.71 ± 5.39
≥85	31.43 ± 4.53	13.33 ± 3.32	31.43 ± 4.53	9.52 ± 2.86	7.14 ± 2.60	20.00 ± 3.90	32	30.48 ± 4.49
MODERATE								
Both sexes								
75-79	16.34 ± 2.60	15.35 ± 2.54	5.94 ± 1.66	3.47 ± 1.29	19.47 ± 2.87	11.39 ± 2.23	20	9.90 ± 2.10
80-84	21.19 ± 3.33	22.52 ± 3.40	8.61 ± 2.28	7.28 ± 2.11	19.29 ± 3.33	15.89 ± 2.98	21	13.91 ± 2.82
≥85	15.33 ± 2.94	24.67 ± 3.52	12.67 ± 2.72	7.33 ± 2.13	13.24 ± 2.91	25.33 ± 3.55	36	24.00 ± 3.49
Male								
75-79	15.58 ± 4.13	11.69 ± 3.66	3.90 ± 2.21	3.90 ± 2.21	14.93 ± 4.35	6.49 ± 2.81	5	6.49 ± 2.81
80-84	18.18 ± 4.75	13.64 ± 4.22	4.55 ± 2.56	4.55 ± 2.56	21.05 ± 5.40	13.64 ± 4.22	7	10.61 ± 3.79
≥85	13.33 ± 5.07	17.78 ± 5.70	6.67 ± 3.72	6.67 ± 3.72	5.26 ± 3.62	17.78 ± 5.70	7	15.56 ± 5.40
Female								
75-79	16.80 ± 3.34	17.60 ± 3.41	7.20 ± 2.31	3.20 ± 1.57	21.95 ± 3.73	14.40 ± 3.14	15	12.00 ± 2.91
80-84	23.53 ± 4.60	29.41 ± 4.94	11.76 ± 3.49	9.41 ± 3.17	18.07 ± 4.22	17.65 ± 4.13	14	16.47 ± 4.02
≥85	16.19 ± 3.59	27.62 ± 4.36	15.24 ± 3.51	7.62 ± 2.59	16.33 ± 3.73	28.57 ± 4.41	29	27.62 ± 4.36
SEVERE/EXTREME								
Both sexes								
75-79	5.45 ± 1.60	14.85 ± 2.50	2.97 ± 1.19	3.47 ± 1.29	12.11 ± 2.37	4.95 ± 1.53	9	4.46 ± 1.45
80-84	8.61 ± 2.28	17.22 ± 3.07	9.93 ± 2.43	4.64 ± 1.71	21.43 ± 3.47	8.61 ± 2.28	12	7.95 ± 2.20
≥85	21.33 ± 3.34	36.00 ± 3.92	17.33 ± 3.09	16.00 ± 2.99	45.59 ± 4.27	18.00 ± 3.14	30	20.00 ± 3.27
Male								
75-79	3.90 ± 2.21	11.69 ± 3.66	3.90 ± 2.21	2.60 ± 1.81	7.46 ± 3.21	3.90 ± 2.21	4	5.19 ± 2.53
80-84	4.55 ± 2.56	13.64 ± 4.22	10.61 ± 3.79	4.55 ± 2.56	14.04 ± 4.60	7.58 ± 3.26	3	4.55 ± 2.56
≥85	22.22 ± 6.20	20.00 ± 5.96	15.56 ± 5.40	11.11 ± 4.68	28.95 ± 7.36	17.78 ± 5.70	7	15.56 ± 5.40
Female								
75-79	6.40 ± 2.19	16.80 ± 3.34	2.40 ± 1.37	4.00 ± 1.75	14.63 ± 3.19	5.60 ± 2.06	5	4.00 ± 1.75
80-84	11.76 ± 3.49	20.00 ± 4.34	9.41 ± 3.17	4.71 ± 2.30	26.51 ± 4.84	9.41 ± 3.17	9	10.59 ± 3.34
≥85	20.95 ± 3.97	42.86 ± 4.83	18.10 ± 3.76	18.10 ± 3.76	52.04 ± 5.05	18.10 ± 3.76	23	21.90 ± 4.04

% ± SE: Specific prevalence ± standard error. Life activities domain was non-applicable for 37 individuals. UAC: Understanding and communication; GAP: Getting along with people; LAC: Life activities; GAR: Getting around; PSO: Participation in society; SCA: Self-care.

**Table 4:** Prevalence OR and 95% confidence interval of extreme/severe disability cases by age and sex

		UAC	GAR	SCA	GAP	LAC	PSO	Summary Index
*Men*	Age (75-70)							
	80-84	1.18 [0.23, 6.03]	1.19 [0.44, 3..21]	2.93 [0.73, 11.81]	1.79 [0.29, 11.02]	2.02 [0.62, 6.58]	2.02 [0.46, 8.80]	0.87 [0.19, 4.03]
	>85	7.05** [1.82, 27.22]	1.89 [0.69, 5.18]	4.54* [1.11, 18.57]	4.69 [0.87, 25.24]	5.05* [1.60, 15.95]	5.33* [1.34, 21.29]	3.36 [0.93, 12.21]
*Women*	Age (75-70)							
	80-84	1.95 [0.74, 5.16]	1.24 [0.61, 2.52]	4.22* [1.09, 16.41]	1.19 [0.31, 4.55]	2.10* [1.05, 4.23]	1.75 [0.61, 5.02]	2.84 [0.92, 8.80]
	>85	3.88* [1.65, 9.13]	3.71** [2.02, 6.82]	8.98** [2.58, 31.29]	5.30* [1.91, 14.75]	6.33** [3.34, 11.97]	3.72* [1.50, 9.24]	6.73** [2.46, 18.43]
*Both*	Sex (Male)							
	Female	1.40 [0.75, 2.62]	1.99* [1.22, 3.24]	0.96 [0.51, 1.83]	1.51 [0.71, 3.25]	2.37** [1.41, 3.99]	1.18 [0.62, 2.23]	1.48 [0.77, 2.87]
	Age (75-70)							
	80-84	1.67 [0.72, 3.83]	1.24 [0.70, 2.21]	3.60* [1.36, 9.51]	1.39 [0.48, 4.04]	2.10* [1.15, 3.82]	1.83 [0.77, 4.29]	1.89 [0.78, 4.62]
	>85	4.60** [2.23, 9.48]	3.12** [1.86, 5.22]	6.87** [2.75, 17.19]	5.16 [2.16, 12.36]	6.00** [3.44, 10.48]	4.16** [1.94, 8.91]	5.23 [2.39, 11.41]

Reference category in parenthesis. UAC: Understanding and communication; GAP: Getting along with people; LAC: Life activities; GAR: Getting around; PSO: Participation in society; SCA: Self-care.

* *p *< .01, ** *p *< .001

Prevalence varied across WHO-DAS II disability domains, sex and age (Table 3). Disability across age in each domain was higher among women (mild: 40.32 ± 2.76% vs. 37.23 ± 3.53%; moderate: 18.41 ± 2.18% vs. 10.11 ± 2.20%; severe/extreme: 11.75 ± 1.81% vs. 7.45 ± 1.91%). Both sexes scored similarly in UAC, with a higher prevalence of mild disability plus a higher prevalence of severe/extreme disability for individuals aged 85 years and over as compared to younger age groups (21.33 ± 3.34%, OR = 4.71, 95% CI = 2.29-9.70). Insofar as GAR was concerned, there was a noticeable difference between men and women, with a higher prevalence of mild disability among men across all age groups (34.57 ± 3.47% vs. 24.13 ± 2.41%, OR = 2.56, 95% CI 1.75-2.56) and a high prevalence of severe/extreme disability among women aged 85 years and over (51.25 ± 4.47% vs. 20.00 ± 5.96%, OR = 3.71, 95% CI 2.03-6.76). There was a high prevalence of no-problem and mild disability in SCA (58.45 ± 2.20%; 23.46 ± 1.89%), particularly among men (OR = 1.61, 95% CI 0.99-2.63). However, prevalence of disability was higher among the oldest women, while men of the same age group displayed a higher proportion of SCA disability-free individuals (51.11 ± 7.45% vs. 35.24 ± 4.66%, OR = 1.92, 95% CI 0.94-1.92). The highest proportion of disability-free individuals was found for the GAP domain (77.14 ± 1.87%), with no age- or sex-specific pattern. In contrast, the LAC domain showed the highest prevalence of severe/extreme disability (24.68 ± 2.00%). This prevalence was particularly high for women aged 85 years and over, as compared to men of the same age (52.04 ± 5.05% vs. 28.95 ± 7.36%, OR = 5.15 95% CI 3.19-8.32). Prevalence of PSO disability was similar for men and women, except for the prevalence of the disability-free category, which was highest in men of 75 to 79 (72.73 ± 5.09% vs. 57.60 ± 4.42%).

In summary, differences in disability prevalence between the sexes seem to be limited to GAR (mobility) and SCA (personal activities of daily living) with women demonstrating higher difficulties in both cases. Prevalence varied significantly across disability domains. The GAR and LAC disability areas concentrated the highest proportions of individuals with moderate to extreme disability. Finally, the number of individuals with severe/extreme disability based on the sex and age distribution of Spanish population for 2009 (age ≥75) [3] was 390,000 (95% CI 338,000 - 442,000).

## Discussion

This study is among the few population-based surveys reporting disability prevalence assessed according to the ICF framework [28,29]. Our results provide a novel view of disability among the Spanish elderly population. Based on WHO-DAS II assessment and ICF disability categories, our study estimated the prevalence of mild, moderate, and severe/extreme disability for those aged 75 years and above. Prevalence rates of severe/extreme disability equalled 10.14%, which works out to a number of 390,000 individuals older than 75 in Spain meeting the case definition used in this study. While most participants displayed some degree of disability (2 out of every 3), the proportion of severely disabled individuals was approximately one tenth of the study sample. These figures are in line with prior studies conducted on European populations using various approaches to the assessment of disability [6,30]. Given the mixed-rural and urban sample in the present study, we should comment on the impact of habitat on dementia. In a recent re-analysis of studies on the prevalence of dementia in Spain, suburban populations showed higher prevalence of Alzheimer's disease while vascular dementia was higher among suburban populations. The pattern was not replicated among urban-mixed populations and there is a paucity of evidence for rural populations [18].

The age-standardised prevalence of disability found in our study was considerably higher than the one reported by the 1999 National Disability Survey for the same age groups [3] (64% vs. 46% of persons aged ≥ 75 with some disability). The fact that our study population was consisted of samples from nine different locations may in part be a protection against selection bias, although our study sample cannot be considered representative of the country's population. Moreover, it should be noted that participants were recruited from the Central-Northern half of the country, which registers lower mortality from all causes [31] and higher longevity and quality of life than does Southern Spain [32]. It should be noted, however, that the presence of dementia cases among participants with non-computable disability data might have reduced our prevalence figures (cf., Alzheimer's type dementia is one of the major disability determinants in the elderly [33]). In addition, cognitive function of participants with non-computable WHO-DAS II was probably slightly inferior than for those with computable scores. Only 25 among the 41 participants with non-computable WHO-DAS II had computable MEC scores, and among those, cognitive function was significantly lower (23.58 ± 10.01 vs. 27.66 ± 6.18; Wilcoxon W, p = .033). These differences are of moderate-low magnitude and within standard criteria for computable scores [34].

The highest functioning level was found for GAP with little variation due to age, suggesting that basic social skills are preserved until very late in life. PSO on the other hand, which evaluates more advanced social skills, showed greater deterioration with age. A similar pattern was observed for SCA and LAC, as these are related to basic and instrumental activities of daily living, respectively. Men and women differed in GAR and LAC domains. Activities in both areas require high cognitive and motor functioning suggesting that the higher prevalence of age-related musculoskeletal and mental disorders (particularly arthritis, osteoporosis and dementia; see Table S1; online additional file 1) may underlie the difference in GAR and LAC performance between women and men.

Multi-dimensional assessment of disability (activities and participation) based on WHO-DAS II opens up a more comprehensive view of disability. The performance of WHO-DAS II as an instrument to inform activities and participation was satisfactory. However, specific aspects of the scale need to be developed further. For instance, the low acceptance of the sexuality item, suggest that this content (among others that are not applicable to all elderly population) needs to be reworded. In addition, WHO-DAS II does not assess the causes of disability. Therefore it is not highly useful to develop strategies to remediate disability. In this respect, ICF core sets represent a recent expansion of ICF framework for the assessment of disease-specific factors of disability. Core sets are composed of clusters of items that are distinctively affected under specific health conditions.

Previous studies using the WHO-DAS II have focused on younger and more specific clinical populations, particularly psychiatric patients [10,35-37]. Our study illustrates the usability of the scale in population-based studies with elderly participants. The WHO-DAS II allows assessing concurrently various aspects of disability, and could be used as the basis for identifying complex relations between disability, and medical, personal, social and environmental factors [38].

The development of methodological strategies for the assessment of disability is highly relevant to clinical epidemiology. Until recently, instruments measuring disability have emphasized clinical profile over behavioral, social and environmental factors [39]. More specifically, the validity of classic measures (e.g., Katz Index, Barthel Index) could be tested against ICF-based approaches to assessment (e.g., ICF checklist) in order to determine the relative contribution of personal, environmental and social factors on individuals' performance.

A study of this nature requires ICF-based instruments that are yet to be developed that would incorporate a relevant core set of items relevant to the characteristics of the disability of the population studied. For instance, this approach has been found effective in assessing the rehabilitation of patients with stroke [40]. The level of morbidity found in elderly populations (Table S1; online additional file 1) suggest the necessity of a generic core set for elderly people allowing an in-depth assessment that would facilitate tailoring services for a particular individual. Our findings are descriptive in nature. Therefore, no social policy guidelines can be drawn from them. Nonetheless, our results are consistent with setting the prevention and remediation of severe/extreme disability in mobility and life activities as a priority for service planning. Strategic actions would require a more thorough examination of disability causes, which are currently the focus of our team.

## Conclusions

Although a number of studies assessing disability in various settings have used the ICF system and the WHO-DAS II, they have been rarely used as the basis for screening and assessment in a population-based study on the prevalence of disability. This approach may be superior to more classical means of determining functional status (e.g., ADL, motor and sensory functioning), which do not allow a multi-faceted appraisal of disability. Our study shows that individuals perform differently in the various areas of disability assessed. Advanced social skills and instrumental activities of daily living, as assessed through the PSO and LAC domains of WHO-DAS II, demonstrated a higher deterioration with age as opposed to other areas of disability. Women tended to perform poorer in areas requiring high motor functioning, as is the case for the GAR and LAC disability domains. The level of specificity achieved by using ICF disability levels and WHO-DAS II may provide essential information for a more comprehensive development of services matching the needs of elderly people.

## List of abbreviations

ADL: Activities of daily living; CI: Confidence interval; GAP: Getting along with people; GAR: Getting around; ICF: International Classification of Functioning a Disability; LAC: Life activities; MMSE: Manitoba Medical Services Foundation; OR: Odd ratio; PSO: Participation in society; SCA: Self-care; UAC: Understanding and communication; WHO-DAS II: World Health Organization-Disability Assessment Schedule 2^nd ^edition.

## Competing interests

This project was partially funded by a research contract in support of the project "Epidemiological Study of Dementia in Spain" signed by the Pfizer Foundation and Carlos III Institute of Health. Funding agencies did not affect the design, implementation, or reporting of this study in any way.

## Authors' contributions

JVO: Conducted the analyses, and wrote the first version of the draft. JPC: Designed the study and wrote various sections of the manuscript. MSM, PS, FSS, FRP, FR, RM, PMM, IM, JGB, JGO, FJG, GFM, FBP, AB, JAI, and JLB: Contributed equally to the study design, data collection, and manuscript writing. All authors read and approved the final manuscript.

## Pre-publication history

The pre-publication history for this paper can be accessed here:

http://www.biomedcentral.com/1471-2458/11/176/prepub

## Supplementary Material

Additional file 1 **General and specific prevalent health conditions and morbidity**. Table S1 showing general and specific prevalent health conditions and morbidity Click here for file
