# Supplementary material for: Prevalence of disability in a composite ≥75 year-old population in Spain: A screening survey based on the International Classification of Functioning
Source: BMC Public Health. 2011 Mar 23;11:176. doi: 10.1186/1471-2458-11-176 (PMC3070655; doi:10.1186/1471-2458-11-176)
Supplement: Additional file 1 — General and specific prevalent health conditions and morbidity. Table S1 showing general and specific prevalent health conditions and morbidity [file 1471-2458-11-176-S1.DOCX]

**Table S1**

General and specific prevalent health conditions and morbidity, % (*n*)

|  | Women | Men | Both |
| --- | --- | --- | --- |
| *General* |  |  |  |
| Circulatory | 67.0 (211) | 64.4 (121) | 66.0 (332) |
| Musculoskeletal | 55.9 (876) | 33.0 (62) | 47.3 (238) |
| Ophthalmological | 37.1 (117) | 37.2 (70) | 37.2 (187) |
| Mental | 35.1 (110) | 19.3 (36) | 29.2 (146) |
| Endocrinological | 29.8 (94) | 27.1 (51) | 28.8 (145) |
| Respiratory | 11.1 (35) | 37.8 (71) | 21.1 (106) |
| Neurological | 13.0 (41) | 23.4 (44) | 16.9 (85) |
| Immunological | 2.5 (8) | 3.2 (6) | 2.8 (14) |
| Infectious | 4.4 (14) | 5.3 (10) | 4.8 (24) |
| Neoplasm | 14.0 (44) | 14.4 (27) | 14.1 (71) |
| *Specific* |  |  |  |
| Hypertension | 56.2 (177) | 48.4 (91) | 53.3 (268) |
| Depression | 20.0 (63) | 5.9 (11) | 14.7 (74) |
| Diabetes | 15.2 (48) | 13.8 (26) | 14.7 (74) |
| Smoking | 1.3 (4) | 26.6 (50) | 10.7 (54) |
| Dementia | 9.5 (30) | 6.4 (12) | 8.3 (42) |
| *Morbidity, M (SD)* | 4.7 (3.1) | 5.0 (3.2) | 4.8 (3.1) |

*Note*. Information retrieved through a physician-administered 51-disease checklist of prevalent diseases in elderly people. Health conditions and disease categories follow ICD-10. Sources: medical file, medical documents held by the examinees, and examinees’ self- and proxy report.
